# Melasolv™: a potential preventive and depigmenting agent for the senescence of melanocytes

**DOI:** 10.3389/fmolb.2023.1228640

**Published:** 2024-01-04

**Authors:** Yeonju Cho, Suh-Yeon Choi, Hyunjung Choi, Mira Ham, Kyu-Han Kim

**Affiliations:** Basic Research & Innovation Division, Research and Innovation Center, Amorepacific, Yongin-si, Republic of Korea

**Keywords:** Melasolv^TM^, depigmenting, melanocyte, senescence, anti-aging

## Abstract

**Introduction:** Senescent melanocytes are major contributors to age-related changes in the skin, highlighting the contribution to skin aging. Moreover, prolonged photodamage, such as that caused by UV exposure, can result in melanin accumulation and accelerated melanocyte senescence, thereby exacerbating aging. Melasolv™ is a substance that induces potent depigmentation effects and exhibits low toxicity. The present study aimed to investigate the potential effect of Melasolv™ on senescent melanocytes.

**Methods:** We profiled the transcriptomics of Melasolv™-treated melanocytes and identified the possible mechanism of action (MOA) and targets using connectivity mapping analysis. We identified differentially expressed genes in response to treatment with Melasolv™ and validated the data using quantitative real-time PCR. Moreover, we performed an *in vitro* β-gal assay in senescent melanocytes for further validation.

**Results:** Melasolv™ reduced β-gal and melanin levels in senescent melanocytes. Moreover, the identified MOAs are associated with anti-aging and anti-senescence effects.

**Discussion:** Our findings clearly indicate that Melasolv™ not only exhibits anti-senescent properties but can also potentially alleviate melanin accumulation in senescent cells. These findings could have far-reaching implications in the treatment of age-related photodamaged skin conditions, such as senile lentigo and melasma.

## 1 Introduction

Melanocytes synthesize melanin to protect the skin from deleterious effects. However, irregular or hyperproduction of melanin causes dark spots on the skin, leading to melasma, senile lentigo, and freckles, which are common cosmetic problems. In addition to melanin synthesis, as part of the neuroendocrine system, melanocytes also possess sensory abilities. Melanocytes secrete various hormones, neuropeptides, and neurotransmitters in response to environmental stressors, thereby maintaining cutaneous homeostasis (Chen et al., 2022). Notably, melanocytes are sentinel immune cells; they perceive changes in the epidermis through their dendritic structures and mediate immune responses in the skin epidermis (Gasque and Jaffar-Bandjee, 2015). Skin aging studies have reported that melanocyte senescence is the major cause of age-related changes in the skin. Moreover, immunohistochemistry analysis shows that melanocytes positive for p16INK4a, a senescence marker, accumulate upon facial aging (Waaijer et al., 2016). These melanocytes induce paracrine senescence and transmit telomere dysfunction to the neighboring cells (Victorelli et al., 2019).

Several skin-lightening compounds, such as hydroquinone, ascorbic acid, and phenylethyl resorcinol, have been developed to reduce melanin synthesis (Zhao et al., 2022). Among these compounds, Melasolv™ has received considerable attention owing to its potent depigmentation effect and low toxicity (Kang et al., 2003; Lee et al., 2017). Melasolv™, a 3,4,5-trimethoxycinnamate thymol ester synthesized by conjugating 3,4,5-trimethoxycinnmic acid with thymol, strongly inhibits melanin synthesis in melan-a cells, primary normal human melanocytes, and human skin equivalents. A recent double-blind clinical study demonstrated that the application of cosmetic products containing 0.1% Melasolv™ to type III-V skin of women living in Southeast Asia led to an improvement in the brightness of pigmented spots (Kim et al., 2021). In addition, treatment with Melasolv™ leads to depigmentation by activating autophagy-induced melanosome degradation (Park et al., 2020).

Several commercially used depigmentation agents induce physiological benefits beyond the conventional anti-melanogenesis efficacy to the skin. For example, cosmetic formulations containing tranexamic acid and alpha-arbutin display both anti-angiogenic and depigmenting effects (Pereira et al., 2020). Furthermore, bakuchiol exhibits depigmenting efficacy and reduces the signs of skin aging (Dhaliwal et al., 2019). This multifunctionality, where compounds can have more than one molecular target, is a basic property of many therapeutic small molecules and serves as a principle for drug repurposing.

Among the different approaches available for identifying new potential mechanisms of action (MOAs) of a compound, signature matching is one of the most commonly used strategies. It is based on comparing the gene expression profiles of interest with the gene expression profiles of known drugs via the connectivity map (CMap). CMap is a large-scale public database that contains over 1.5 million gene expression profiles of approximately 5,000 small molecules tested in multiple cell types. Highly ranked CMap drugs may have similar MOAs to the compound of interest, which has been widely applied in various pharmacological research for drug repurposing (Qu and Rajpal, 2012). Therefore, the present study aimed to investigate the potential novel functions of Melasolv™ beyond its whitening properties based on the drug repurposing concept of CMap. Subsequently, the preventive effect of Melasolv™ on the senescence was experimentally validated.

## 2 Materials and methods

### 2.1 Reagent

Melasolv™ was synthesized by AMOREPACIFIC, as previously described (Kang et al., 2003).

### 2.2 Cell culture

Human epidermal melanocytes derived from the neonatal foreskin of moderately pigmented donors (African-American males) were purchased from Cascade Biologics (Portland, OR, United States) and cultured in Medium 254 (Thermo Fisher Scientific, Waltham, MA, United States) supplemented with a human melanocyte growth supplement (Thermo Fisher Scientific) under humidified 5% CO_2_ atmosphere. Cells from passages 2 to 5 were used in this study. For transcriptional profiling, melanocytes were treated with 10 μg/mL Melasolv™ for 1 day.

Senescence in melanocytes was induced as described in our previous study (Choi et al., 2018). In brief, melanocytes with the indicated concentration in Figure were exposed to sub-cytotoxic doses (20 mJ/cm^2^) of UVB twice, with a 24-h interval in between. Melasolv™ treatment was administered at the time of the first UVB exposure. After 2 weeks of culture, SA-β-galactosidase activity and melanin contents were analyzed.

### 2.3 RNA-seq analysis

Total RNA was extracted using the TRIzol reagent (Thermo Fisher Scientific) according to the manufacturer’s instructions. cDNA from the total RNA was fragmented, and the Illumina TruSeq Stranded Total RNA Library Prep kit (Illumina, San Diego, CA, United States) was used for library preparation. RNA-seq was conducted by Macrogen (Seoul, Korea) using the NovaSeq 6,000 platform (Illumina) (GEO ID: GSE229700). Triommomatic version 0.38 (Bolger et al., 2014) was used for trimming and quality control, and HISAT2 version 2.1.0 (Kim et al., 2015) and Bowtie2 version 2.3.4.1 (Langmead and Salzberg, 2012) were used to map the reads to the human genome reference UCSC hg19 database. Raw read counts were quantified using STRING Tie version 1.3.4d (Pertea et al., 2015). Differentially expressed genes (DEGs) were then identified using the DEseq2 R package (Love et al., 2014). DEGs were defined using the following criteria: |log_2_FoldChange (log_2_FC)| ≥ 1 and adjusted *p* < 0.05.

### 2.4 Volcano plot and heatmap visualization

Volcano plots for the expression levels of Melasolv™-treated melanocytes and melanocyte inducing transcription factor (MITF) targets were generated using the ggplot2 R package, and heatmaps were visualized using the pheatmap package in R. MITF targets of Melasolv™ were extracted using the DoRothEA R package (Garcia-Alonso et al., 2019). Targets with high confidence levels (A, B, and C) were selected.

### 2.5 Enrichment analysis

Gene Ontology (GO) analysis of DEGs was performed using Metascape version 3.5 (Zhou et al., 2019). Representative terms were selected from the top GO terms or pathways (*p* < 0.01). The Gseapy Python package (Fang et al., 2023) was used for the Gene Set Enrichment Analysis (GSEA) using the libraries Reactome_2022 and MSigDB_hallmarks_2020. We ranked Melasolv™-induced gene expression profiles based on the log2FC value.

### 2.6 Connectivity analysis

The CMap library, a collection of gene expression profiles of drug-induced human cancer cells, has been widely used in drug repurposing studies (https://clue.io/data/CMap2020#LINCS2020) (Lamb et al., 2006). In this study, the PharmacoGx R package (Smirnov et al., 2016) was used to determine connectivity scores. The CMap dataset provides gene expression profiles at different normalization levels ranging from 1 to 5. Level 5 data that represent transcriptomic signatures (i.e., differential gene expression in response to perturbation, e.g., chemical compounds, gene knockdown, gene knockout, and overexpression) were used. Only the signatures with a perturbation time exceeding 24 h were used as reference profiles. The input data for connectivity mapping analysis comprised upregulated and downregulated DEGs induced by Melasolv™ treatment. DEGs were compared with reference gene signatures using a pattern-matching algorithm based on the non-parametric rank-ordered Kolmogorov-Smirnov statistic. Compounds with significant positive connectivity scores may have an MOA similar to that of Melasolv™, and therefore, these compounds were used as reference compounds to explore possible novel biological functions of this drug.

### 2.7 Text mining

Chilibot (Chen and Sharp, 2004) was used to estimate the relationship between the target genes of compounds with top connectivity scores and aging/senescence. Chilibot enables the retrieval of biological relationships between genes and biological processes using natural language processing integrated with biomedical knowledge. The results consisted of weights for interactive relationships, which reflect the number of abstracts obtained from PubMed; relationships with weights higher than 15 were considered. The selected interactive relationships between target genes and aging/senescence were visualized using Cytoscape (v.3.8.2, https://cytoscape.org/).

### 2.8 Quantitative real-time PCR (RT-qPCR)

The extracted total RNA was reverse-transcribed to generate cDNAs using a Superscript Reverse Transcriptase II kit (Thermo Fisher Scientific). RT-qPCR was performed using the TaqMan Universal Master PCR mix and TaqMan Gene Expression Assays (Thermo Fisher Scientific) using an ABI7500 FAST real-time PCR system (Applied Biosystems, Thermo Fisher Scientific). The measurement of the relative expression of mRNAs was carried out using probes detailed in Supplementary Table S1. Human glyceraldehyde 3-phosphate dehydrogenase (GAPDH; 4333764F; Applied Biosystems) was also amplified and used to normalize variations in cDNA quantities.

### 2.9 Senescence-associated β-galactosidase (SA-β-gal) assay

The mammalian β-gal assay kit was purchased from Thermo Fisher Scientific. Two weeks after treatment with Melasolv™, proteins from melanocytes were extracted using a mammalian protein extraction reagent (M-PER; Thermo Fisher Scientific). After reaction with β-gal assay reagent, the absorbance at 405 nm was measured using Synergy H2 microplate reader (BioTek, Winooski, VT, United States). SA-β-gal staining was performed using a β-gal staining kit (K145501; Invitrogen, Carlsbad, CA, United States) according to the manufacturer’s instructions. Images of five to ten random fields were obtained using an optical light microscope (Nikon Eclipse TS100, Tokyo, Japan) at a magnification of 200x.

### 2.10 Melanin measurement

Cells were counted and pellets w containing melanin were dissolved in 1 N NaOH and incubated for 30 min at 60 °C. The melanin contents were determined by measuring the absorbance at 450 nm using a Synergy H2 microplate reader (BioTek) and compared with that of a standard curve of synthetic melanin (Sgima).

### 2.11 Measurement of DNA damage

Melanocytes were exposed to 20 mJ/cm^2^ of UVB twice with a 24-h interval, and Melasolv™ treatment was administered at the time of first UVB exposure. Genomic DNAs were extracted from melanocytes using the Wizard Genomic DNA Purification Kit (Promega, Madison, WI, United States), according to the manufacturer’s instructions. To quantitatively measure the damage of DNA, the formation of 8-hydroxydeoxyguanosine, one of the oxidative DNA damage byproducts, was detected using OxiSelect™ Oxidative DNA Damage ELISA kit (Cell Biolabs Inc., San Diego, CA, United States) according to the manufacturer’s instructions.

### 2.12 Statistical analysis

All statistical data are presented as the mean ± SD from three independent experiments. A two-tailed Student’s *t*-test was used to analyze differences between the two groups. *p < 0.05, **p < 0.01.

## 3 Results

### 3.1 Transcriptomic analysis of Melasolv™-treated melanocytes

**FIGURE 1:**
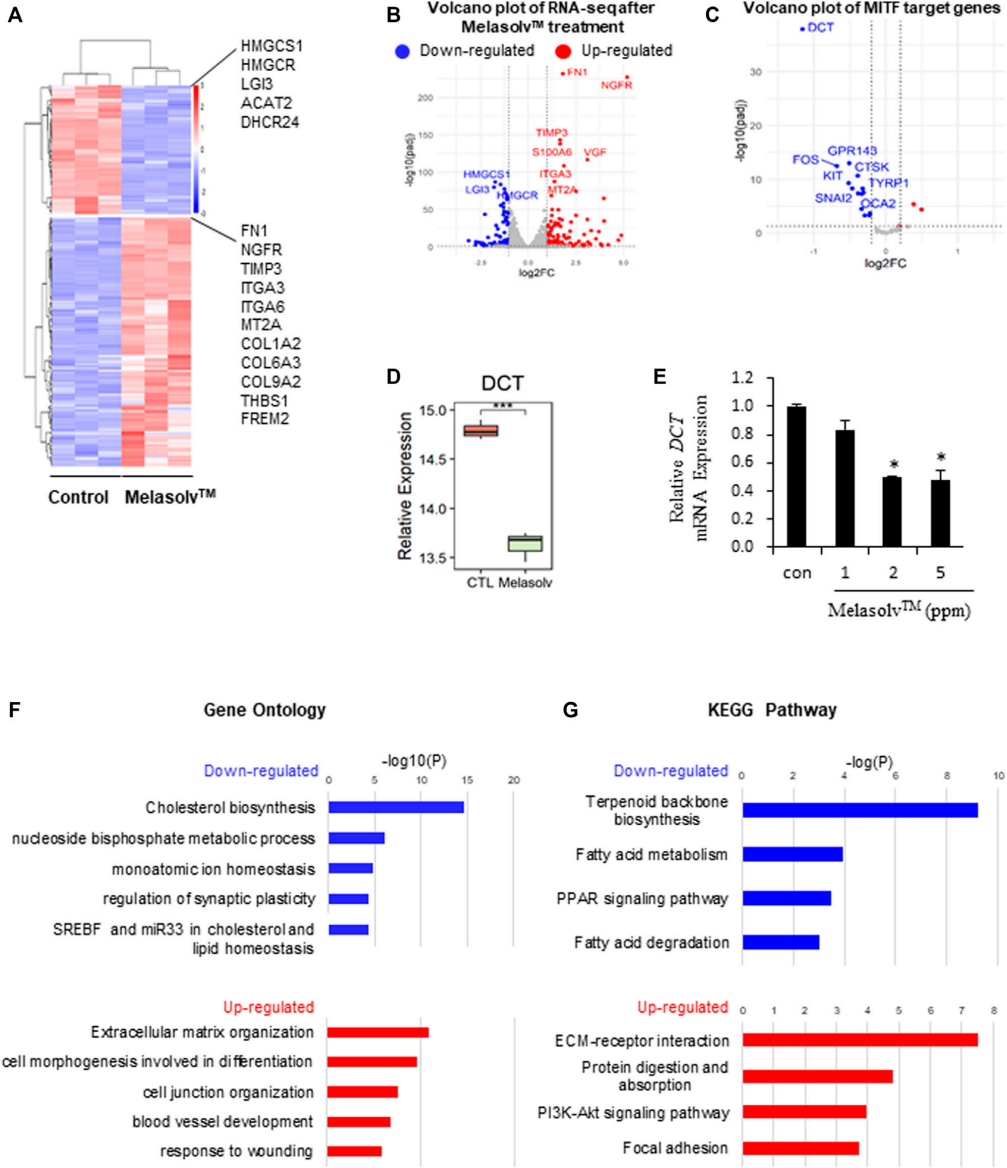
RNA-seq analysis of human primary melanocytes treated with 10 μg/mL Melasolv™. **(A)** Heatmap of differentially expressed genes (DEGs) following Melasolv™ treatment. **(B)** Volcano plot of RNA-seq data. **(C)** Volcano plot of target genes of MITF, a transcription factor associated with melanogenesis. Significantly upregulated and downregulated genes are highlighted in red and blue, respectively. **(D)** Relative expression level (log2 (Deseq2 normalized value + 1)) of dopachrome tautomerase (DCT) in control and Melasolv™-treated melanocytes. **(E)** Quantitative real-time PCR analysis of the relative mRNA expression of *DCT*. Data are presented as the mean ± SD of three independent experiments. *p < 0.05, unpaired Student’s *t*-tests. **(F)** Gene Ontology and **(G)** Kyoto Encyclopedia of Genes and Genomes analysis of the DEGs.

We performed differential gene expression analysis to identify genes that were differentially expressed following Melasolv™ treatment in human melanocytes. A total of 255 DEGs were identified (|log2FC| > 1 and adjusted *p*-value <0.05), including 168 upregulated and 87 DEGs in Melasolv™-treated melanocytes compared to the control (Figures 1A,B and Supplementary Table S2). We validated the mRNA expression of selected DEGs using RT-qPCR (Supplementary Figure S1) and confirmed that the RNA-seq data is consistent. In our analysis, we first examined the changes in the expression of genes related to the depigmentation function of Melasolv™. Notably, the mRNA expression of dopachrome tautomerase (*DCT*), an enzyme that initiates melanogenesis by catalyzing tyrosine conversion, was significantly reduced (Figures 1D,E). Moreover, analysis of the collection of target genes of transcription factors obtained using DoRothEA tools (Garcia-Alonso et al., 2019) revealed that the target genes of MITF are significantly downregulated following treatment with Melasolv™ (Figure 1C and Supplementary Table S3).

To gain further insight into the biological functions of the identified DEGs, we performed enrichment analyses (Figures 1D,E). GO and Kyoto Encyclopedia of Genes and Genomes (KEGG) analyses revealed that the genes upregulated following Melasolv™ treatment were related to cell differentiation, wound-healing responses, and extracellular matrix organization. Moreover, the downregulated genes in Melasolv™-treated cells were significantly enriched in processes such as cholesterol biosynthesis. These analyses indicate that Melasolv™ induces significant changes in genes associated with various biological processes. This result suggests that the potential biological effects of Melasolv™ may extend beyond its well-known anti-melanogenesis function.

### 3.2 Connectivity mapping analysis

The connectivity mapping approach is widely used to uncover unknown functions of drugs (Musa et al., 2018). The CMap database provides large-scale drug perturbation data, including transcriptomic profiles of reference signatures of drugs and their known MOAs and target genes. To infer potential MOAs associated with Melasolv™, we performed a connectivity mapping analysis. We used DEGs induced by Melasolv™ as input queries for the CMap database. Each reference signature was ranked according to its connectivity score, with the top-ranked signatures showing the strongest correlation with the Melasolv™ signature. The analysis revealed several compounds with high connectivity scores and potential MOAs, including PIM kinase inhibitors, TRPV agonists, retinoid receptor agonists, mTOR inhibitors, HDAC inhibitors, RAF inhibitors, and BCL inhibitors (Table 1). These compounds have previously been shown to have therapeutic effects in various disease conditions and may provide insights into the potential MOA of Melasolv™.

**TABLE 1:** Top-ten compounds with high connectivity scores.

Compound	Target	MOA	Score	*p*-value
GDC-0349	PIK3CA	PIM kinase inhibitor	6.674e-01	5.060e-04
Disulfiram	ALDH2	TRPV agonist/DNA methyltransferase inhibitor/Aldehyde dehydrogenase inhibitor	6.666e-01	5.160e-04
CD-1530	RARG	Retinoid receptor agonist	6.576e-01	5.090e-04
Sirolimus (Rapamycin)	FKBP1A/MTOR/PIK3CA/PIK3CD/PIK3CG	mTOR inhibitor	6.544e-01	4.730e-04
Emodin	CSNK2A1	11-beta-HSD1 inhibitor	6.537e-01	4.620e-04
Vorinostat	HDAC6/HDAC1/HDAC2/HDAC8/HDAC3	HDAC inhibitor	6.514e-01	4.940e-04
MK-1775	WEE1	WEE1 kinase inhibitor	6.505e-01	4.840e-04
PLX-4720	BRAF/FGFR1/FLT1/FLT3/FLT4/KDR/KIT/DDR2/PDGFRB/RAF1/RET	RAF inhibitor	6.483e-01	9.360e-04
TW-37	MCL1/BCL2/BCL2L1	BCL inhibitor	6.455e-01	5.830e-04
Rotenone	ND1	Mitochondrial complex I inhibitor	6.365e-01	4.790e-04

**FIGURE 2:**
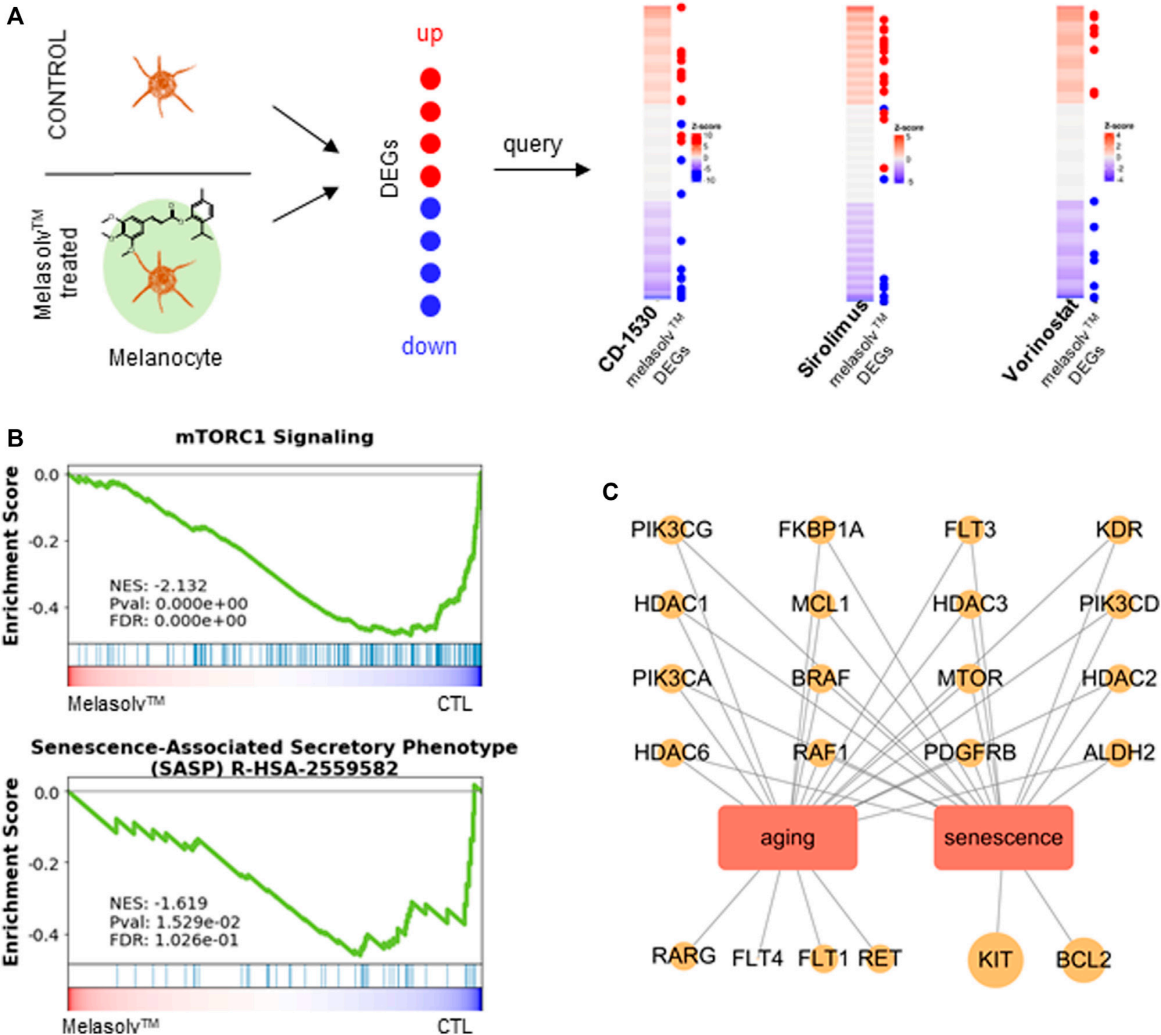
Connectivity mapping analysis of Melasolv™. **(A)** The query signature (Melasolv™ DEGs) was compared against reference profiles (CMap) to compute connectivity. For top compounds with high connectivity scores, a distinctive pattern was exhibited where upregulated genes appeared at the top and downregulated genes appeared at the bottom of the reference profile. **(B)** Gene Set Enrichment Analysis (GSEA), performed using aging-associated terms (mTORC1 signaling and SASP), demonstrates the transition in gene expression from untreated melanocytes to Melasolv™-treated melanocytes. Significant enrichment of senescence and the mTOR signaling pathway was observed within the gene pool that was downregulated upon the treatment with Melasolv™. The normalized enrichment score (NES), *p-*value, and false discovery rate (FDR) are indicated in the insert. **(C)** The relationship network of target genes upregulated by Melasolv™ was analyzed from the connectivity mapping with aging/senescence.

Among the top compounds identified by connectivity mapping analysis, CD-1530 (Thacher et al., 2000), sirolimus (Wang et al., 2017; Chung et al., 2019; Blagosklonny, 2022), and vorinostat (McIntyre et al., 2019) exhibit well-known anti-aging functions. Visualization of the query gene mapped onto reference signatures showed that many upregulated DEGs following Melasolv™ treatment were mapped near the top of the ranked reference signature list, whereas downregulated DEGs appeared at the bottom of the reference signature list (Figure 2A and Supplementary Table S4). We validated the mRNA expression of selected genes (*ACAT2*, *MVD*, and *HMGCR*) that significantly contributed to the high connectivity with anti-aging-associated compounds using RT-qPCR (Supplementary Figure S2). In addition, the results of GSEA showed that the senescence-associated secretory phenotype (SASP) and mTOR signaling pathways were significantly enriched among the downregulated genes (Figure 2B). These pathways play important roles in skin aging and senescence (Sharma and Padwad, 2019). Notably, in the enrichment analysis, we observed that genes upregulated by the treatment with Melasolv™ were significantly associated with processes of cellular differentiation (Guo et al., 2022) and wound-healing ability (Khalid et al., 2022). These processes are well-documented to diminish with aging. Conversely, cholesterol biosynthesis was enriched among downregulated genes, and previous research has indicated that cholesterol can induce melanogenesis and the release of cyclic adenosine monophosphate (cAMP) in epidermal melanocytes (Schallreuter et al., 2009). Furthermore, activation of the cAMP pathway is associated with the expression of p27 and p16 and the loss of E2F activity, which are markers of cellular senescence in human melanocytes (Haddad et al., 1999). These findings imply that Melasolv™ may could potentially inhibit the biological processes associated with aging. To further validate the link between the connectivity mapping results and aging and senescence, we used a text-mining approach using Chilibot (Chen and Sharp, 2004). From the PubMed database, we obtained the frequency of studies that related to each of the top-ten target genes involved in aging/senescence. These frequencies are depicted as dot sizes in the network shown in Figure 2C. Of these 30 genes, 22 genes were associated with aging/senescence. Taken together, the results of connectivity mapping, GSEA, and literature mining analyses suggest that Melasolv™ treatment may prevent the aging/senescence of melanocytes.

### 3.3 Anti-senescence effect of Melasolv™

**FIGURE 3:**
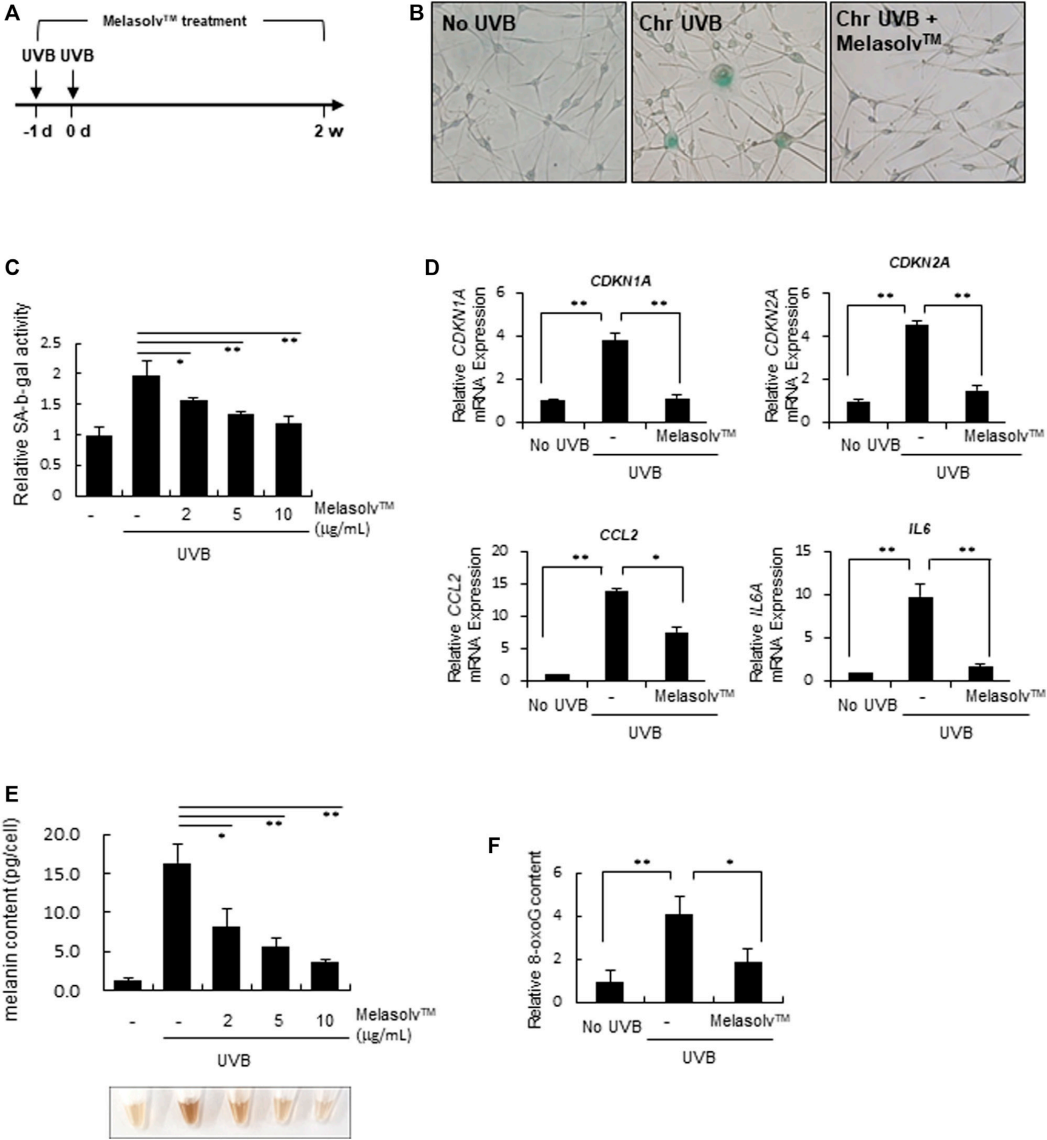
Preventive effect of Melasolv™ on senescence **(A)** Scheme of *in vitro* experiments for treatment with Melasolv™ in a senescent melanocyte model. (B, and **(C)** After 14 days of incubation, SA-β-gal assay was performed by staining **(B)** or by measuring the activity **(C)**. **(D)** The indicated mRNA expression in melanocytes subjected to the treatment of 5 μg/mL Melasolv™ or no treatment was quantitatively assessed using real-time PCR. **(E)** The melanin content in each pellet was visualized (bottom) or determined by measuring the absorbance at 450 nm (up). **(F)** After second UVB exposure, the 8-oxoG content in melanocytes subjected to the treatment of 5 μg/mL Melasolv™ or no treatment was measured using ELISA. Data are presented as the mean ± SD of three independent experiments. *p < 0.05, unpaired Student’s *t*-tests.

We subsequently examined the preventive effect of Melasolv™ on the senescence of melanocytes *in vitro*. We used a previously established melanocyte senescence model in which human epidermal melanocytes were exposed twice to 20 mJ/cm^2^ UVB over a 24-h interval (Figure 3A) (Choi et al., 2018). At the time of first UVB exposure, cells were treated with Melasolv™. As previously shown, chronic UVB exposure induced a senescent phenotype, which included an increased number of flattened and enlarged cells and high production of pH-dependent SA-β-Gal after 2 weeks of cultivation. In contrast, treatment with Melasolv™ significantly blocked the senescent phenotypes (Figures 3B,C). This preventive effect was further assessed by analyzing the mRNA expression of the cell cycle regulator genes *CDKN2A* and *CDKN1A,* encoding cyclin-dependent kinase inhibitors, and two selected SASP-related genes, *IL6* and *CCL8*. As shown in Figure 3D, treatment with Melasolv™ significantly reduced the upregulated expression of these genes. In addition, treatment with Melasolv™ effectively protected melanocytes against UVB-induced DNA damage. These results indicate that Melasolv™ can broadly block the damaging effects of UV, subsequently preventing the senescence process.

Melanin content is increased in senescent melanocytes (Choi et al., 2018; Park et al., 2023); therefore, we assessed changes in the melanin content in response to treatment with Melasolv™. Treatment with Melasolv™ mitigated the increased melanin content in senescent melanocytes (Figure 3E). Our results indicate that Melasolv™ not only exerts a significant depigmenting effect but also exhibits a potent efficacy in preventing senescence, suggesting its potential as an effective treatment for addressing age-related skin pigmentation concerns.

## 4 Discussion

The CMap database offers an extensive repository of drug-induced gene expression profiles, facilitating drug repurposing by revealing the MOA of both existing and novel compounds, predicting target genes, and enhancing our understanding of biological processes (Musa et al., 2018). In the present study, we used connectivity mapping analysis to predict the potential MOA of Melasolv™, which was previously recognized only for its effect on depigmentation.

Our *in silico* findings revealed that the leading compounds exhibiting high connectivity scores to the gene expression signatures of Melasolv™-treated samples had MOAs linked to anti-aging/senescence pathways (Table 2). For example, the PIM kinase inhibitor exhibits anti-inflammatory effects via regulation of the NF-κB and JAK/STAT signaling pathways. Similarly, retinoic acid is known to alleviate photoaging and cellular senescence induced by the loss of MTI-MMP. Additionally, mTOR inhibition is a well-known anti-aging mechanism, whereas HDAC inhibitors exert their anti-aging effects through epigenetic regulation. BRAF and BCL inhibitors exert anti-aging effects through the reversal of cellular senescence. Overall, these results indicate that Melasolv™ might exert its anti-aging effects by modulating multiple pathways. While the exact signaling pathway and mechanism remain unknown, CMap analysis suggested the possible anti-senescence effects of Melasolv™. Notably, we confirmed a potent efficacy of Melasolv™ in preventing senescence using *in vitro* experiments, supporting the phenotypic assumption.

**TABLE 2:** Association of aging/senescence with the MOA of the top connectivity score compounds.

MOA	Relation to aging/senescence	Ref
PIM kinase inhibitor	- Anti-inflammatory effect by regulating the NF-κB signaling pathway	Baek et al. (2020), Malone et al. (2020), Clements and Warfel (2022)
	- Associated with JAK/STAT signaling induced by cytokines	
	- Interconnected signaling pathway with PI3K/mTOR	
Retinoid receptor agonist	- All-trans retinoic acid treatment ameliorates cellular senescence induced by loss of MTI-MMP	Mukherjee et al. (2006), Gutierrez-Fernandez et al. (2015)
	- Retinoid alleviates photodamage (wrinkling, sallowness, and mottled hyperpigmentation)	
MTOR inhibitor	- Rapamycin (mTOR) complex can reduce senescence and levels of markers of aging in human skin (reduction in p16INK4A protein levels and increase in collagen VII protein levels)	Wang et al. (2017), Chung et al. (2019), Blagosklonny (2022)
HDAC inhibitor	- HDAC inhibitors act as anti-aging drugs	Pasyukova and Vaiserman (2017), McIntyre et al. (2019)
RAF inhibitor	- Oncogenic BRAF induces melanocyte senescence	Dhomen et al. (2009)
BCL inhibitor	- BCL inhibitor reverses irradiation-induced senescence	Yosef et al. (2016), Sharma and Padwad (2019)
	- BCL inhibitor induces apoptosis in senescent cells	

A previous study on senescent melanocytes reported that the observed upregulation of melanin is a consequence of melanosome transport dysfunction rather than an enhanced melanogenesis process [Ref]. However, our transcriptomic data analysis in Melasolv™-treated melanocytes revealed that Melasolv™ does not influence the expression of genes associated with melanosome transfer, such as *RAB27A*, *MYO5A*, and *MLPH*. This result indicates that Melasolv™ is not a specific inhibitor of melanosome transfer. Therefore, we propose that Melasolv™ might effectively prevent melanocyte senescence by obstructing the deleterious effects induced by UV exposure. Furthermore, our findings highlight that Melasolv™ has the capacity to broadly mitigate the DNA damage induced by UV, thereby inhibiting the process of senescence Despite these insights, more comprehensive studies are required to understand the mechanisms underlying the anti-aging function of Melasolv™.

As the skin ages, senescent melanocytes accumulate, forming mottled pigmentation. Functional and morphological phenotypes in melanocytes are altered by chronic exposure to photodamage, such as UV stimuli, and during aging. Subsequently, the accumulated damage leads to the senescence of melanocytes (Choi et al., 2018; Hughes and Bishop, 2022). Immunohistochemical analysis has demonstrated that melanocytes are the major contributors of senescent cells in individuals after middle age, which largely contribute to the aging phenotype, such as the appearance of facial wrinkles, and a perceived acceleration of the aging process (Waaijer et al., 2016; Kim et al., 2022). The SASP secreted by aged melanocytes induces telomere dysfunction in a paracrine manner and impairs keratinocyte proliferation (Victorelli et al., 2019). Moreover, irregular hyperpigmentation, such as senile lentigo and melasma, is a characteristic of older sun-exposed skin (Kang et al., 2021). Repeated UV irradiation induces melanin accumulation and melanocyte senescence (Bandyopadhyay and Medrano, 2000; Choi et al., 2018). Overall, melanin synthesis and senescence are important factors in the treatment of photoaged skin. The present study demonstrated that Melasolv™ can effectively reduce the levels of melanin and β-gal in senescent melanocytes, thus providing a potential solution for photoaging in skin. Therefore, Melasolv™ can be considered an effective substance for treating skin conditions related to photoaging by targeting melanin synthesis and senescence.

## Data Availability

The datasets presented in this study can be found in online repositories-https://www.ncbi.nlm.nih.gov/geo/query/acc.cgi?acc=GSE229700.

## Abbreviations

DCT, dopachrome tautomerase; FDR, false discovery rate; GO, Gene Ontology; GSEA, Gene Set Enrichment Analysis; MOA, mechanism of action; SASP, senescence-associated secretory phenotypes.
