# Supplementary figures and images for: Melasolv™: a potential preventive and depigmenting agent for the senescence of melanocytes
Source: Front Mol Biosci. 2024 Jan 4;10:1228640. doi: 10.3389/fmolb.2023.1228640 (PMC10935553; doi:10.3389/fmolb.2023.1228640)

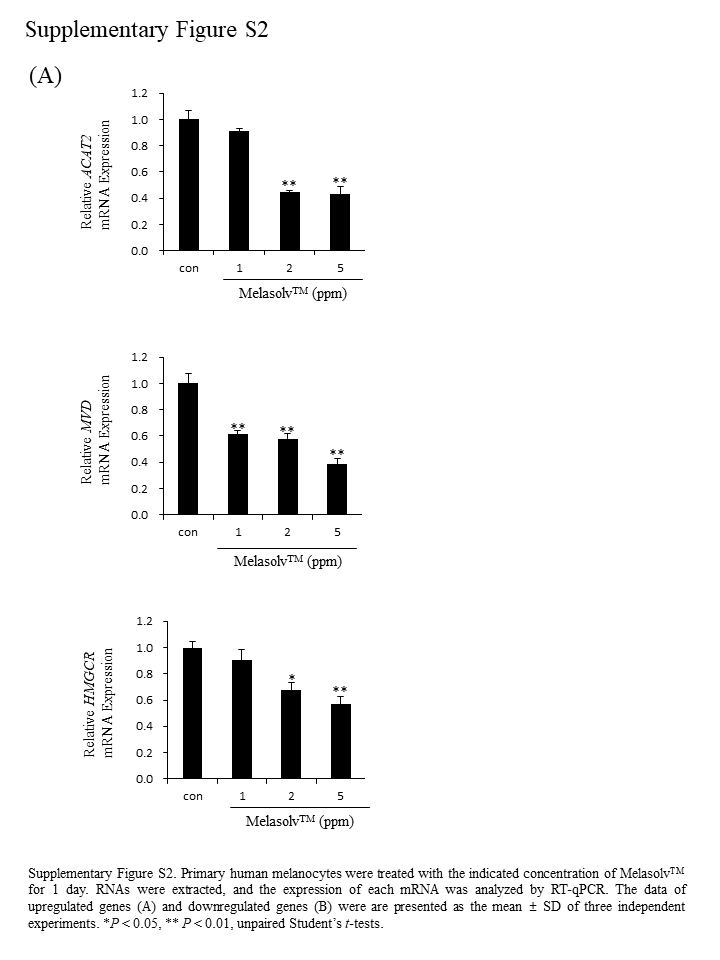

Supplement: Supplementary file 3 [file Image2.TIF]

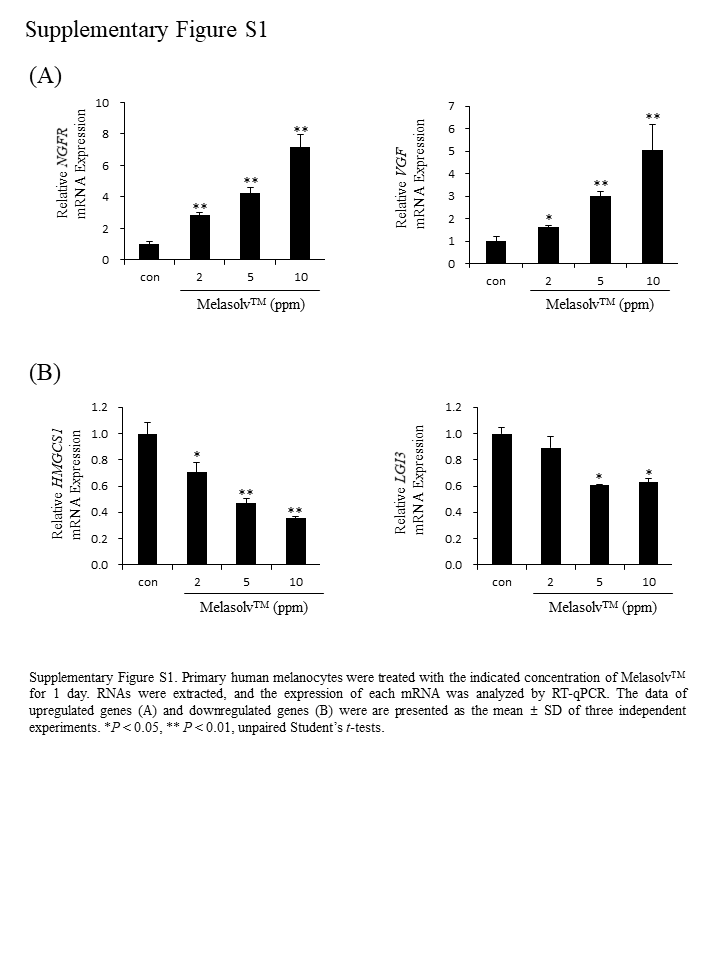

Supplement: Supplementary file 4 [file Image1.TIF]
